# 遗传性血栓性血小板减少性紫癜5例临床分析

**DOI:** 10.3760/cma.j.issn.0253-2727.2023.01.008

**Published:** 2023-01

**Authors:** 昕波 吕, 杰 殷, 丹青 孔, 竑 田, 云 李, 琦 曲, 健 苏, 丽娟 曹, 霞 白, 自强 余, 兆钺 王, 德沛 吴, 长耿 阮

**Affiliations:** 苏州大学附属第一医院血液科，江苏省血液研究所，国家血液系统疾病临床医学研究中心，国家卫生健康委员会血栓与止血重点实验室，血液学协同创新中心，苏州大学省部共建放射医学与辐射防护国家重点实验室，苏州 215006 Jiangsu Institute of Hematology, National Clinical Research Center for Hematologic Disease, NHC Key Laboratory of Thrombosis and Hemostasis, The First Affiliated Hospital of Soochow University, Collaborative Innovation Center of Hematology, State Key Laboratory of Radiation Medicine and Protection, Soochow University, Suzhou 215006, China

**Keywords:** 紫癜，血栓性血小板减少性, ADAMTS13蛋白质, 诊断，鉴别, Purpura, thrombotic thrombocytopenic, ADAMTS13 protein, Diagnosis, differential

## Abstract

**目的:**

报道5例遗传性血栓性血小板减少性紫癜（cTTP）患者的临床表现及实验室检查特点，结合文献资料探讨cTTP的临床诊治方法。

**方法:**

分析患者发病年龄、疾病表现、个人史、家族史、误诊情况等临床资料，观察血浆输注疗效和器官功能评估等治疗结局；结合血浆ADAMTS13活性测定及ADAMTS13基因突变分析，探讨cTTP临床表现、治疗结局与ADAMTS13基因突变间的相互内在联系。

**结果:**

5例CTTP患者中男1例，女4例，发病年龄分别为1、9、12、19、31岁，主要临床表现为血小板明显减少、贫血及神经系统受累表现；既往多被疑诊为免疫性血小板减少症；2例女性患者因妊娠诱发，其他病例存在感染诱因。所有病例均检出ADAMTS13基因突变，基因突变区域与患者临床表现及器官损伤程度之间存在内在联系。治疗性和预防性血浆输注是有效的治疗方法。

**结论:**

cTTP临床表现具有明显异质性，易被误诊而延误治疗。ADAMTS13活性检测和基因突变分析是重要诊断依据，预防性血浆输注可有效控制疾病发作。

## Abstract (en)

**Objective:**

To report the clinical manifestations and laboratory features of five patients with congenital thrombotic thrombocytopenic purpura（cTTP）and explore its standardized clinical diagnosis and treatment along with a review of literature.

**Methods:**

Clinical data of patients, such as age of onset, disease manifestation, personal history, family history, and misdiagnosed disease, were collected. Treatment outcomes, therapeutic effects of plasma infusion, and organ function evaluation were observed. The relationship among the clinical manifestations, treatment outcomes, and ADAMTS13 gene mutation of patients with cTTP was analyzed. Additionally, detection of ADAMTS13 activity and analysis of ADAMTS13 gene mutation were explored.

**Results:**

The age of onset of cTTP was either in childhood or adulthood except in one case, which was at the age of 1. The primary manifestations were obvious thrombocytopenia, anemia, and different degrees of nervous system involvement. Most of the patients were initially suspected of having immune thrombocytopenia. Acute cTTP was induced by pregnancy and infection in two and one case, respectively. ADAMTS13 gene mutation was detected in all cases, and there was an inherent relationship between the mutation site, clinical manifestations, and degree of organ injury. Therapeutic or prophylactic plasma transfusion was effective for treating cTTP.

**Conclusion:**

The clinical manifestations of cTTP vary among individuals, resulting in frequent misdiagnosis that delays treatment. ADAMTS13 activity detection in plasma and ADAMTS13 gene mutation analysis are important bases to diagnose cTTP. Prophylactic plasma transfusion is vital to prevent the onset of the disease.

遗传性血栓性血小板减少性紫癜（cTTP）是临床上较为少见的疾病，多以出血和血小板减少为首发表现，常被误诊为免疫性血小板减少症（ITP）而不能得到及时治疗[1]。近年来，随着对cTTP认识的提高以及检测手段的完善，尤其是二代基因测序技术的普及，cTTP的临床诊治水平显著提高[2]。我们报告5例cTTP患者的临床特点及诊疗经过并进行文献复习，旨在加深对cTTP的认识、提高相关疾病的诊疗水平。

## 病例资料

例1，女，22岁。因“停经3个月，血小板进行性减少2个月伴头痛2周”入院。患者10余年前有鼻出血及血小板减少史。半年前查血常规示PLT 13×10^9^/L、HGB 80 g/L，2周前因少量阴道出血伴阵发性前额胀痛、皮肤散在出血点及瘀点就诊。门诊头颅CT未见明显异常；血常规：PLT 19×10^9^/L，HGB 72 g/L，血涂片红细胞明显大小不等、易见晚幼红。病程中未发生神志异常，无肢体运动和感觉异常。其父母为近亲结婚。体检：神志清，精神欠佳；全身皮肤可见散在瘀点，全身浅表淋巴结无肿大，肝脾触诊不满意；宫底位于脐耻之间。PLT 11×10^9^/L，HGB 73 g/L，网织红细胞0.22％，破碎红细胞2％；尿蛋白2+；总胆红素35.9 µmol/L，丙氨酸氨基转移酶（ALT）198 U/L，天冬氨酸氨基转移（AST）202 U/L，乳酸脱氢酶（LDH）1378 U/L，肌酐（Cr）133 µmol/L，血肌钙蛋白15.62 ng/L。Coombs试验阴性。产科超声提示宫内单胎存活。心电图无异常。残余胶原结合试验法（R-CBA）检测血ADAMTS13活性0％，抑制物阴性。诊断cTTP、早孕。血浆置换共2 d，总量3 500 ml。复查PLT 46×10^9^/L，R-CBA法检测ADAMTS13活性100％，LDH 619.7 U/L；5 d后复查PLT 344×10^9^/L、HGB 100 g/L，患者一般情况明显好转；18 d后复查PLT 20×10^9^/L，因患者坚持继续妊娠，当日输注新鲜血浆800 ml，5 d后复查PLT 187×10^9^/L；此后多次预防性血浆输注。孕29周时因重度子痫前期、胎儿宫内窘迫而提前终止妊娠。分娩后恢复情况良好，定期接受预防性血浆输注。二代基因测序：ADAMTS 13 chr9:136289572 exon3 c.304C>T p.Arg102Cys（MP区），纯合错义突变。

例2，女，31岁，因“阵发性头晕、呕吐伴言语不清1个月”入院。患者妊娠5个月时出现呕吐、言语不清、四肢无力，外院查WBC 14.16×10^9^/L、HGB 65 g/L、PLT 9×10^9^/L；超声示宫内孕，横位（考虑死胎）。外院测ADAMTS13活性<5.0％、ADAMTS13抗体833.77 µg/L；诊为TTP，行血浆置换7次、糖皮质激素治疗（不详）并行引产术，血小板计数恢复正常。两周前PLT 19×10^9^/L，再次行血浆置换3次。病程中无明显头晕乏力及活动性出血。否认父母近亲结婚史。入院查体：右锁骨、左手背处瘀斑，余无明显异常。血常规：WBC 18.59×10^9^/L，HGB 110 g/L，PLT 275×10^9^/L，外周血破碎红细胞<2％；Coombs试验阴性；LDH 249 U/L，ALT 180.5 U/L，AST 33.8 U/L，Cr正常；心电图无异常；超声示“右侧股总静脉置管周围血栓形成”。ADAMTS13活性0％，ADAMTS13抑制物阴性（R-CBA法）。入院后血小板计数持续下降至10×10^9^/L，血浆输注治疗3 d（血浆总量1 550 ml），次日PLT 171×10^9^/L。二代基因测序：ADAMTS 13 c.2923G>A, p.Gly1308Asp（CUB2区）、NM_139025.4 c.3645-1G>A（CUB1区）复合杂合突变，确诊为cTTP。近期未行血浆输注，血小板计数正常。

例3，女，14岁，因“血小板减少13年，右侧肢体活动不灵2月余”入院。患者1岁时诊断“ITP”，予糖皮质激素及静脉注射免疫球蛋白治疗有效。后反复发生皮肤瘀点、瘀斑及血小板减少，未治。3个月前左上肢瘀斑、血小板减少，骨髓巨核细胞158个伴成熟障碍；外院诊断ITP，泼尼松治疗无效。2个月前出现言语不能及右侧肢体活动不灵，PLT 19×10^9^/L，HGB 79 g/L；血浆ADAMTS13活性0％，ADAMTS13自身抗体19.98 U/mL（弱阳性）；二代基因测序：ADAMTS13 c.1192C>T, p.Arg398Cys（T1区）和c.304C>T, p.Arg102Cys（MP区）突变。外院给予血浆置换、静脉注射免疫球蛋白、抗CD20单抗等治疗。2周前出现大肠埃希菌败血症，血常规HGB 86 g/L、PLT 12×10^9^/L；ADAMTS13活性0.67％，ADAMTS13抗体阴性。经抗感染、糖皮质激素激素、血浆输注治疗后病情好转。否认父母近亲结婚。入院查体：神情，轻度贫血貌，右上肢可见陈旧性瘀斑，余无明显异常。血常规PLT 150×10^9^/L，HGB 93 g/L，WBC 4.85×10^9^/L；白蛋白37.5 g/L，LDH 470.4 U/L，Cr 49.4 µmol/L；Coombs试验阴性；心电图正常；脑电图示两半球见中量中幅慢波出现，各区慢频带能级增高。头颅磁共振血管造影（MRA）：多发脑梗死；ADAMTS13活性100％（R-CBA法），抑制物阴性。入院诊断：cTTP，多发性脑梗死。入院1周后PLT 61×10^9^/L，予以血浆输注2次（总量1400 ml），复查PLT 276×10^9^/L。2周后血小板计数再次下降，再次输注血浆，好转后出院。当地继续每2周1次预防性输注血浆600 ml，血小板计数和病情稳定。

例4，男，29岁，因“血小板减少20余年，发热、腹痛腹泻2 d”入院。20年前因皮肤出血诊断为ITP，治疗不详。1月前进食冰水后出现腹痛，外院WBC 13.8×10^9^/L、HGB 158 g/L、PLT 45×10^9^/L；尿潜血+++，蛋白质++，总胆红素140.6 µmol/L，直接胆红素13.0 µmol/L，间接胆红素127.6 µmol/L，Cr 124.9 µmol/L；骨髓象：粒系比例略低，胞浆可见颗粒增多，红系比例增高，巨核细胞成熟障碍。予糖皮质激素、止血、护胃治疗后好转出院。20 d前出现流涕伴明显头痛，头颅CT未见异常，血常规示WBC 19.14×10^9^/L、HGB 127 g/L、PLT 11×10^9^/L；ADAMTS13活性0％（R-CBA法），抑制物阴性。疑诊cTTP。输注新鲜血浆2次，复查PLT 116×10^9^/L。入院前2 d患者受凉后发热、腹痛腹泻，入住本院。否认既往其他疾病史，患者父母系近亲婚配。入院查体：体温37.8 °C，全身皮肤及巩膜轻度黄染，无瘀点瘀斑、无水肿，肝脾肋缘下未触及。血常规：WBC 15.14×10^9^/L，HGB 139 g/L，PLT 18×10^9^/L；LDH 451 U/L，AST 24.8 U/L，ALT 43.3 U/L，总胆红素33.4 µmol/L。血浆ADAMTS13活性0％、抑制物阴性（R-CBA法）；Coombs试验阴性；心电图正常。二代基因测序：ADAMTS13 chr9:136289551; exon3;c.283G>C; p.Ala95Pro纯合错义突变（MP区）。诊断：cTTP伴肠道感染。给予血浆输注2次（每次400 ml），糖皮质激素减量使用。出院后间断不规则输注血浆，血小板计数正常。

例5，女性，19岁，因“间断发热伴乏力8 d”外院就诊。患者8 d前受凉后出现发热，伴头晕、恶心、呕吐，查血常规HGB 72 g/L、PLT 4×10^9^/L，血片可见破碎红细胞，LDH 4962 U/L。考虑TTP。予4次血浆置换并输注洗涤红细胞，地塞米松、抗感染等治疗后病情好转。病程中患者神志清楚，无发热、咳嗽、咯痰。未婚未育，平素月经正常，否认父母近亲结婚。查体：无明显阳性体征。血常规：WBC 5.93×10^9^/L、HGB 79 g/L、PLT 90×10^9^/L；ALT 41 U/L，LDH 240 U/L，Cr正常。血浆ADAMTS13活性0％（R-CBA法）、抑制物阴性；Coombs试验阴性，初步诊断TTP。继续血浆置换、新鲜血浆输注及甲泼尼龙治疗，血小板恢复正常，病情好转后出院。出院后甲泼尼龙减量使用，间断输注血浆，血小板计数持续正常。二代基因测序：ADAMTS13 chr9:136307582; exon17、c.2031C>A, p.Tyr677Ter纯合无义突变（S区）。确诊cTTP。

## 讨论与文献复习

cTTP也称Upshaw-Schulman综合征，是一种常染色体隐形遗传性疾病。因ADAMTS13基因突变致ADAMTS13合成、释放障碍，血浆ADAMTS13活性部分或完全缺乏，微血管内血小板-血管性血友病因子（VWF）异常聚集，导致严重血小板减少、微血管病性溶血性贫血、相关脏器缺血性损伤及功能障碍[2]。cTTP年发病率为1～2/百万，但由于本病临床表现具有明显异质性及相关检测手段不足，其实际发生率可能被低估[3]。挪威的一项研究显示cTTP的年发病率可达16.7/百万[4]。

cTTP起病年龄不一、病情严重程度不等。可在新生儿期发病，主要表现为严重黄疸、出血、血小板减少，易与新生儿黄疸混淆。早期发病者往往病情危重，存活者常伴有不同程度的器官功能损害。Hager等[5]报道一例出生后即发病的cTTP患儿，存在ADAMTS13基因纯合突变，虽经治疗仍在出生34 h后死亡。近年来国内也有多个新生儿期发病的cTTP案例报道，部分患儿经血浆输注治疗后得以存活[6]–[8]。

儿童期或成年期发病的cTTP患者的临床表现不一，除明显血小板减少外，贫血及其他症状如头痛、嗜睡、腹痛等也不典型，易被忽视。10％的患者可发展为慢性肾功能衰竭[9]。女性cTTP患者常于妊娠期发病，在妊娠前三个月内发生的血栓性微血管病中，cTTP几乎是唯一的病因[10]。Tenison等[11]报道1例反复脑梗死男性成年患者，经ADAMTS13活性测定和基因分析确诊为ADAMTS13基因纯合突变cTTP。诱发因素常包括感染、炎症反应、妊娠等，这提示单独ADAMTS13活性缺乏并不足以引起临床症状发生，炎症因子、血管内皮损伤、补体异常活化等因素共同作用才导致疾病发作[12]。本组患者中女4例、男1例，发病年龄分别为1、9、12、19、31岁。其中2例为妊娠早期诱发疾病发作或加重，其余3例均存在感染等诱发因素。

根据国际血栓与止血协会（ISTH）诊断治疗指南[13]–[14]，TTP诊断依赖于临床表现、血液学变化、血浆ADAMTS13活性和抑制物测定。根据PLASMIC评分，对中高危患者进行ADAMTS13活性及抑制物测定并及时启动治疗；血浆ADAMTS13活性<10％可以确立TTP诊断，抑制物或ADAMTS13相关IgG阳性可诊断为免疫性TTP（iTTP），抑制物阴性则提示cTTP，需行ADAMTS13基因检测明确诊断。

青少年期或成年期发病的cTTP患者常起病隐匿、症状相对较轻或临床表现不典型，易被长期误诊为ITP，导致延误治疗及不同程度器官功能受损[15]。本组病例中，有3例均在确诊前被误诊为ITP，且对常规ITP治疗无反应；从疾病初发到确诊cTTP分别间隔了10、13、20年；且有不同程度的肾功能和脑功能损伤。另外本组有2例患者（例2、例3）外院检出ADAMTS13自身抗体，但我院ADAMTS13抑制物测定阴性，依据治疗反应和基因检测结果确诊为cTTP，提示ADAMTS13自身抗体检测存在假阳性可能。建议对显著血小板减少伴发不能解释的肝、肾、脑损伤患者，尤其是父母为近亲婚配者应考虑cTTP诊断的可能，并与iTTP相鉴别。此外，在妊娠早期发生不明原因的血小板数显著减少或TMA患者首先要考虑cTTP可能。

至今在cTTP患者中已发现超过200种ADAMTS13基因突变；基因突变类型涉及点突变、无义突变、小片段缺失、以及剪切位点突变等，导致酶活性中心异常或截断蛋白形成[16]。通常，涉及ADAMTS13酶活性区域的突变往往发病早，病情较重；而其他功能域突变常发病较迟，病情较轻。本组病例中，例1和例4基因检测均为ADAMTS13蛋白酶活性（MP）区纯合突变，分别为c304C>T, pArg102Cys和c283G>C, pAla95Pro，均属于致病基因突变，已有文献报道；例3为包含MP区c304C>T, pArg102Cys突变在内的复合杂合突变。这3例患者发病较早，也因显著血小板减少而长期被误诊为ITP，其中例3患者因反复微血管血栓形成导致较明显的器官功能损伤。例2患者ADAMTS13基因突变为发生在C端的CUB区双重杂合突变，例5患者ADAMTS13基因突变发生在Spacer区，并不直接影响到该蛋白的酶活性区，可能残留一定水平的酶切活性水平，这2例患者起病较晚（分别为19、31岁）且例2是在首次妊娠时发作。所以对cTTP进行ADAMTS13基因检测不仅可以确立诊断，而且可以根据基因突变类型预测患者病情和预后。

cTTP治疗原则主要包括祛除诱因、补充缺乏的ADAMTS13、保护器官功能[14],[17]。感染和炎症是引起cTTP发作的主要诱因，应积极抗感染治疗祛除诱因，减少炎症因子对血管内皮损伤及活化血小板。补充缺乏的ADAMTS13是目前cTTP治疗和预防的主要方法，主要采用新鲜（冰冻）血浆输注，病情严重者可行血浆置换。ISTH指南[14]推荐血浆输注剂量为每次10～15 ml/kg；ADAMTS13体内半衰期为2～3 d，预防性治疗时推荐血浆输注每1～3周1次，可根据血小板计数变化以及有无新的器官功能异常进行调整；按需治疗时可每日输注至病情缓解。重组ADAMTS13（rADAMTS13）已进入Ⅲ期临床研究，为cTTP患者按需治疗和预防治疗提供了更为便捷、有效的治疗选择，推荐预防治疗剂量为每次40 IU/kg每1～3周1次。由于cTTP患者病情反复发作，部分患者重要器官功能损伤（如肾脏损害、脑血管及脑功能损伤、心功能损伤等），需要在替代治疗的同时密切观察并及时治疗器官功能损伤，以降低疾病相关致残率和死亡率[18]。本组有2例患者未规律性预防治疗，血小板计数正常，提示应根据个人病情特点选择个体化治疗方案。

女性cTTP患者再次妊娠会导致疾病复发或病情复杂化，对母婴双方均会引起不良影响。推荐从妊娠开始至产后4～6周进行血浆输注预防治疗，输注剂量每次10～15 ml/kg，输注间隔从每2周1次逐渐缩短为每周1次或每日1次，严重者可行血浆置换[19]。监测指标包括血小板计数、LDH、器官功能状况等。如病情控制良好，可以维持妊娠至36～37周分娩；如病情控制不良或伴发其他妊娠相关的并发症如妊高症、子痫、HELLP综合征等需要及时终止妊娠。

近年来多种新型治疗手段应用于TTP治疗。Caplacizumab单抗可通过封闭VWF A1区阻断VWF与血小板GPIb结合，抑制VWF-血小板聚集体的形成[20]。新型融合蛋白Microlyse具有抗VWF CK区抗体重链可变区片段与尿激酶型纤溶酶原活化剂（uPA）片段的功能，可降解微血管内已经形成的VWF-血小板聚集体微血栓[21]。cTTP作为单基因疾病且具有轻、中度提升ADAMTS13活性即可控制疾病发作的特点，适合采用基因治疗方法治疗本病，有可能成为根治本病的方法。

总之，cTTP临床少见，临床表现在个体间有较大差异，易被长期误诊为其他疾病而延误治疗。全面分析病情、及时进行ADAMTS13活性检测有助于早期诊断，基因突变分析可以确立诊断。除发作期治疗外，预防性血浆输注或rADAMTS13替代治疗对减少cTTP患者疾病发作、阻止器官损伤有重要作用。
